# Quantifying trade-offs between therapeutic efficacy and resistance dissemination for enrofloxacin dose regimens in cattle

**DOI:** 10.21203/rs.3.rs-4166888/v1

**Published:** 2024-04-09

**Authors:** Liton Chandra Deb, Archana Timsina, Suzanne Lenhart, Derek Foster, Cristina Lanzas

**Affiliations:** 1 Department of Population Health and Pathobiology, College of Veterinary Medicine, North Carolina State University, Raleigh, NC, USA; 2 Department of Mathematics, University of Tennessee, Knoxville, TN, USA

**Keywords:** Enrofloxacin dosing regimens in cattle, Antimicrobial drug resistance, Pk-Pd model

## Abstract

The use of antimicrobial drugs in food-producing animals increases the selection pressure on pathogenic and commensal bacteria to become resistant. This study aims to evaluate the existence of trade-offs between treatment effectiveness, cost, and the dissemination of resistance in gut commensal bacteria. We developed a within-host ordinary differential equation model to track the dynamics of antimicrobial drug concentrations and bacterial populations in the site of infection (lung) and the gut. The model was parameterized to represent enrofloxacin treatment for bovine respiratory disease (BRD) caused by *Pastereulla multocida* in cattle. Three approved enrofloxacin dosing regimens were compared for their effects on resistance on *P. multocida* and commensal *E. coli*: 12.5 mg/kg and 7.5 mg/kg as a single dose, and 5 mg/kg as three doses. Additionally, we explored non-approved regimes. Our results indicated that both 12.5 mg/kg and 7.5 mg/kg as a single dose scenario increased the most the treatment costs and prevalence of *P. multocida* resistance in the lungs, while 5 mg/kg as three doses increased resistance in commensal *E. coli* bacteria in the gut the most out of the approved scenarios. A proposed scenario (7.5 mg/kg, two doses 24 hours apart) showed low economic costs, minimal *P. multocida,* and moderate effects on resistant E. coli. Overall, the scenarios that decrease *P. multocida*, including resistant *P. multocida* did not coincide with the scenarios that decrease resistant *E. coli* the most, suggesting a trade-off between both outcomes. The sensitivity analysis indicates that bacterial populations were the most sensitive to drug conversion factors into plasma (*β*), elimination of the drug from the colon (*υ*), fifty percent sensitive bacteria (*P. multocida*) killing effect (*L*_*s*50_), fifty percent of bacteria (*E. coli*) above ECOFF killing effect (*C*_*r*50_), and net drug transfer rate in the lung (*γ*) parameters.

## 1. Introduction

Antimicrobial drugs are used in food-producing animals to treat, control, and prevent infections. In the United States, the Food and Drug Administration (FDA) is responsible for approving antimicrobial drugs intended to be used for food-producing animals^1^. In addition to ensuring the safety and effectiveness of the drug in the target species, the FDA evaluates the drugs based on whether there is reasonable certainty that their use in food-producing animals will not harm human health^1^. For antimicrobial drugs, a specific consideration is whether the drug can cause resistance and how resistance to the drug can impact public health^1^. Recently, FDA proposed a risk assessment approach to evaluate the potential microbial food safety risks associated with new antimicrobial drugs^2^. While the proposed risk assessment is qualitative, FDA encourages the use of modeling approaches that can refine and improve the approach and assumptions incorporated in this risk assessment process^2^.

Ideally, antimicrobial drug use should be optimized to achieve therapeutic efficacy while minimizing the emergence of antimicrobial drug resistance in both pathogens and host-associated commensal bacteria. Dissemination of resistance in the gut bacteria is of particular concern as the gut of food animals is a reservoir for foodborne pathogens^3^. Emergence and further dissemination of resistance depends on multiple factors, including the antimicrobial drug concentrations to which bacteria are exposed, the genetic basis of resistance, and the relative fitness of the resistant organisms^4^. These factors can be explicitly addressed and evaluated in mathematical models. Mathematical models grounded on pharmacokinetics (PK), pharmacodynamics (PD) and microbial growth dynamics can provide a risk assessment of the effects of dose and treatment duration on bacterial dynamics^5^. Overall, only a few mathematical models address the emergence and dissemination of antimicrobial resistance during treatment in food animals^6–8^. It is unknown what is the effect of currently approved dose regimes on the emergence of resistance in off-target bacteria such as commensal gut bacteria.

Bovine respiratory disease (BRD) is one of the most common diseases affecting feedlot cattle, causing high mortality, reduced performance, and subsequently decreased carcass value^9,10^. BRD is also one of the most common indications for giving individual-level treatment to feedlot cattle^9,10^. Enrofloxacin – a veterinary-approved fluoroquinolone- is a common choice for BRD treatment because of its high potency against the gram-negative aerobic bacteria that commonly cause BRD, such as *Mannheimia haemolytica* and *Pasteurella multocida*^11,12^. However, there are concerns that the use of enrofloxacin has contributed to the emergence of fluoroquinolone resistance in some foodborne pathogens, such as *Campylobacter spp*.^13,14^.

The main goal of this project is to study whether there are trade-offs between the treatment efficacy, economic costs, and the level of resistance of pathogenic and enteric commensal bacteria when treating BRD in cattle. We developed and implemented a deterministic mathematical model of within-host *P. multocida* infection and enteric *E. coli*. We simulated different treatment regimens (duration and dosage) of enrofloxacin and evaluated their effects on *P. multocida* infection and resistance, as well as its implications for selecting resistance in off-target bacteria in the gut. Additionally, we performed a sensitivity analysis to identify the PK and PD parameters that influence the most resistance in both the target and off-target bacterial populations.

## 2. Materials and methods

### 2.1 Model description

**Figure 1:**
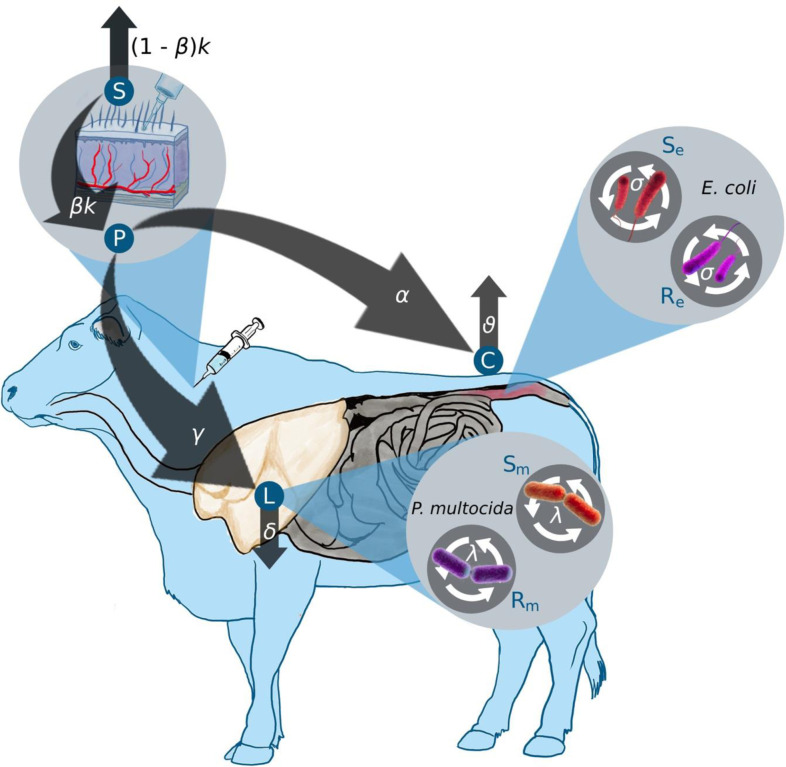
Schematic representation of the mathematical model depicting enrofloxacin concentrations in the gastrointestinal and respiratory tracts of a steer, and its effects on both *E. coli* and *P. multocida* bacteria populations. The compartments *S*, *P*, *L*, and *C* represent antimicrobial drug concentrations in subcutaneous tissue, plasma, lung, and colon, respectively. *S*_*e*_, *R*_*e*_, *S*_*m*_, and *R*_*m*_ represent the susceptible and resistant subpopulation of *E. coli* and *P. multocida*. Parameters are described in text.

A system of ordinary differential equations was developed to track the dynamics of the antimicrobial drug concentrations and the bacterial populations in the lungs and gut. The model flowchart is presented in (Fig. 1).

Enrofloxacin is administered to the subcutaneous compartment *S* with a dose denoted as *S*_0_, given *i* times at regular intervals T. This input process is mathematically represented by the Dirac function *δ*(t-iT). The drug is eliminated from the subcutaneous compartment *S* at a rate of *k*. The antimicrobial drug enters the plasma at a rate of *β*k. Within the plasma, the total concentration of both bound and unbound antimicrobial drug is denoted as *P*. The drug is then transferred from the plasma to the colon and lungs at a net transfer rate of *α* and *γ*, respectively. Finally, the drug levels in the colon *C* and the lungs *L* are eliminated at rates of *υ* and *δ*, respectively. All the mentioned rates apply to both enrofloxacin and its metabolized form, ciprofloxacin, combined.

The bacterial populations, *E. coli* and *P. multocida*, grow logistically with net growth rates of *σ* and *λ*, respectively. The maximum carrying capacity for the *E. coli* and *P. multocida* are denoted as *N*_*emax*_ and *N*_*mmax*_. The net growth rate of a bacterial strain is considered to represent the bacterial fitness at the within host level^15^. We modeled the fitness costs associated with resistance by including a fractional reduction in the net growth rates, represented by *c* and *p* for the resistant *E. coli* (*R*_*e*_) and resistant *P. multocida* (*R*_*m*_) subpopulations, respectively.

The pharmacodynamic effects of enrofloxacin are modeled by a sigmoidal function representing the saturation of the death rate as a function of the antimicrobial drug concentration^5^. We assume that the concentration required to produce half of the maximum death effect is greater for resistant bacteria than sensitive bacteria, hence *C*_*r*50_ > *C*_*s*50_ and *L*_*r*50_ > *L*_*s*50_.

The model described above is represented by the system of differential equations given below:

(1) $$\frac{dS}{dt}=\sum_{i=0}^{n}S_{0}\delta_{0}(t-iT)-KS\;\frac{dP}{dt}=\beta KS-(\alpha +\gamma )P\;\frac{dC}{dt}=\alpha P-\vartheta C\;\frac{dL}{dt}=\gamma p-\delta L\;\frac{dS_{e}}{dt}=\sigma \left(1-\frac{S_{e}+R_{e}}{N_{\text{emax}}}\right)S_{e}-d\frac{c}{C+C_{s50}}S_{e}\;\frac{dR_{e}}{dt}=(1-c)\sigma \left(1-\frac{S_{e}+R_{e}}{N_{\text{emax}}}\right)\;R_{e}-d\frac{c}{C+C_{r50}}R_{e}\;\frac{dS_{m}}{dt}=\lambda \left(1-\frac{S_{m}+R_{m}}{N_{\text{mmax}}}\right)R_{m}-\eta \frac{L}{L+L_{s50}}S_{m}-\phi S_{m}\;\frac{dR_{m}}{dt}=(1-p)\lambda \;\left(1-\frac{S_{m}+R_{m}}{N_{\text{mmax}}}\right)R_{m}-\eta \frac{L}{L+L_{r50}}R_{m}-\phi R_{m}$$

with initial conditions *S* (0) = 0 mg/kg, *P* (0) = 0 mg/kg, *C* (0) = 0 *μg*/*ml*, *L* (0) = 0 *μg*/*ml*, *S*_*e*_ = 400000 colony forming units (CFUs), *R*_*e*_ = 1000 CFUs, *S*_*m*_ = 40000 CFUs, *R*_*m*_ = 1000 CFUs.

### 2.2 Data and model parameterization

The experimental data used to partially parameterize the model were previously described in Foster et al.^16,17^ and was approved by the North Carolina State University Institutional Animal Care and Use Committee. Moreover, Foster et al.^16,17^ affirmed adherence to animal welfare and ARRIVE guidelines in their study, demonstrating meticulous attention to ethical standards and methodological rigor. The data for fitting the PK components of the model were collected from two experimental studies where a cohort of twelve steers were monitored following two approved dosing scenarios: a single dose of enrofloxacin (12.5 mg/kg), and a single dose of enrofloxacin (7.5 mg/kg), both administered subcutaneously. After the subcutaneous administration, enrofloxacin concentration in plasma, colon, and interstitial fluid were measured over time. Additionally, *E. coli* concentration in the gut, measured as CFU/ml, and its minimum inhibitory concentration (MIC) was determined according to the established guidelines of the Clinical and Laboratory Standards Institute^6^. To capture the changes of overall bacteria population in the model, we denoted the sub-population of *E. coli* and *P. multocida* with MICs above the epidemiological cut-off value as resistant (*R*_*e*_ and *R*_*m*_), and those below the cut-off point were defined as susceptible (*S*_*e*_ and *S*_*m*_). The epidemiological cut-off value for enrofloxacin on *E. coli* and *P. multocida* is the same, 0.125 *μg*/*ml*^18,19^.

Parameters that can be derived uniquely from the data are considered identifiable^20,21^. Structural identifiability is a theoretical way to determine whether the within-host ODE model’s parameters are identifiable from the noise-free observations without the actual data^22^. A structurally unidentifiable parameter obtained from fitting is not valid to use in further analysis of the ODE model^20,21,23^. In our model, we investigated the structural identifiability of the parameters before estimating the values of the parameters. We used a user friendly and universally accessible web application COMBOS^24^ for checking structural identifiability. COMBOS uses a Grobner-based computation to check the structural identifiability of the parameters^24^.

To estimate the PK parameters from the data, we used the software Monolix 2020 R1 (Lixoft, Antony, France). We independently fitted the model to the concentration of antimicrobial drug in the plasma, colon, and lung, and susceptible and resistant *E. coli* population in the feces for each steer in the treatment groups. The parameter estimation was done using stochastic approximation expectation maximization (SAEM). SAEM is a technique that combines maximum likelihood estimation with stochastic approximation to estimate conditional expectations^25^. We performed a maximum of 200 Monte Carlo runs each with 10,000 iterations. The parameter *k*, *C*_*s*50_, *L*_*s*50_, *L*_*r*50_ were fixed based on previous studies, as shown in Table 1. In our PK-PD model, we assumed all the parameters had log-normal distributions. Also, we let *σ*, *d*, and *N*_*emax*_ vary across study populations to capture the dynamics of *E. coli* at the individual level. The reason to estimate those parameters is that commensal *E. coli* populations typically vary across individuals and can be highly dynamic^26^. By using the data from Foster et al.^17^, we estimated *β*, *α*, *υ*, *σ*, and *N*_*emax*_. Also, by using data from Foster et al.^16^, we estimated *γ*, *d* and *δ*. Then *N*_*mmax*_, *λ*, *c*, *η*, *p*, and *ϕ* values were calibrated to a scenario in which the disease-causing *P. multocida* bacteria was cleared in the model, effectively achieving disease clearance.

### 2.3 Simulated scenarios

In our study, we simulated the impact of three approved enrofloxacin dosing scenarios and some non-approved proposed scenarios on the treatment efficacy against one of the causative agents of BRD, *P. multocida*. Specifically, in the case of approved scenarios, we simulated 12.5 mg/kg and 7.5 mg/kg as a single dose, and doses of 5 mg/kg given 24 hr apart for three days, all administered subcutaneously. In case of proposed non-approved dosing scenarios, we simulated doses of 12.5 mg/kg, 3.75 mg/kg, 6.25 mg/kg, and 7.5 mg/kg, administered 24 hours apart for two days. Additionally, we examined doses of 4.15 mg/kg, given subcutaneously 24 hours apart for three consecutive days. Our investigation aimed to simulate the effects of these drug dosing regimens and gain insights into their therapeutic efficacy, treatment cost, and bacterial resistance outcomes. Our study assessed the treatment expenses associated with different dosing scenarios. To quantify each treatment scenario’s cost, we considered the average cost of medications and labor and the productivity losses resulting from the treatment process. If the infection was not fully resolved (defined in the model as presence of bacteria in the lung after treatment), we added an additional treatment in our analysis.

### 2.4 Uncertainty and sensitivity analysis

To identify the PK and PD parameters that have the most influence on the bacterial resistance within the lung and gut sites, *R*_*m*_and *R*_*e*_, we performed a variance-based sensitivity analysis. This Sobol sensitivity approach uses model output uncertainty to describe the variance^28,29^. We have chosen to vary the following PK and PD parameter set, q = (*k*, *β*, *α*, *γ*, *υ*, *δ*, *C*_*s*50_, *C*_*r*50_, *L*_*s*50_, *L*_*r*50_). As a model output, we evaluated uncertainty of both the susceptible and resistant of *E. coli* and *P. multocida* bacterial populations. We calculated the first-order Sobol indices and total-order Sobol indices of the parameter set q for the bacterial resistant^30–32^. The basic algorithm of the calculation of the indices is as follows:

- Providing lower and upper bound values for each parameter of q as an input, we obtain N possible values uniformly distributed for each of the parameters of q.
- Taking the distributions of the set of parameters q as an input variable, the within-host model (1) generates N = 2000 set of solutions, also called uncertainty distribution of solutions. In this study, we focus on the uncertainty distribution of states *S*_*e*_(*t*), *R*_*e*_(*t*), *S*_*m*_(*t*), and *R*_*m*_(*t*).
- As mentioned, we are only concerned with calculating the Sobol indices of *R*_*m*_ and *R*_*e*_. Here, we calculated the overall variance of the resistant using deviation from the average value of the time-dependent uniform distribution of the resistant. Let the overall variance of *R*_*m*_ be denoted by $V_{R_{m}}$.
- The first-order variance $V_{S_{i}}$ of *R*_*m*_ is calculated with respect to an individual parameter *q*_*i*_ of the set q.
- The total-order variance $V_{T_{i}}$ of *R*_*m*_ is calculated with respect to all parameters except the individual parameter *q*_*i*_ of the set q.
- The time-dependent first-order Sobol index of the parameter *q*_*i*_ for *R*_*m*_ is calculated with formula $S_{i}=\frac{V_{S_{i}}}{V_{R_{m}}}$ and the time-dependent total-order Sobol index of the parameter *qi* for *R*_*m*_ is calculated with formula $T_{i}=1-\frac{V_{T_{i}}}{V_{R_{m}}}$.
- The sum of the first-order indices of all parameters q such as ∑_*i*_ *S*_*i*_ is less than 1. Similarly, ∑_*i*_ *T*_*i*_ also is less than 1. If *S*_*i*_ or *T*_*i*_ of a parameter is close to 1, then that parameter significantly influences the state *R*_*m*_. On the other hand, if *S*_*i*_ or *T*_*i*_ of a parameter is close to 0, that parameter has low influence on the state *R*_*m*_.
- As the indices *S*_*i*_(*t*) and *T*_*i*_(*t*) of each parameter of set q are time dependent, we plotted the curves of the indices over time T=150 hours
- The curve thus created by the indices of each parameter of q over time 150 hours is integrated to get area using the Riemann sum. These areas help us to compare the level of sensitivity of parameters of set q for *R*_*m*_ for the complete simulation period.
- Similarly, we repeated the similar process of finding Sobol indices and integrated area for *R*_*e*_.

We used the “Sensobal package” of R software version 4.3.1 to calculate the Sobol indices^32,33^. The input bounds for q, the generated uniform distribution of states *S*_*e*_, *R*_*e*_, *S*_*m*_, and *R*_*m*_ as well as the results of Sobol indices of the model parameters q for resistant bacteria *R*_*m*_ and *R*_*e*_ are presented in the results section. We utilized the proposed value for each parameter as given in Table 1 and select upper and lower bounds not more than 3 standard deviations from each parameter of set q. Each parameter’s upper and lower bounds of q are in Table 2

## 3. Results

### 3.1 Simulated cases scenarios

**Figure 2:**
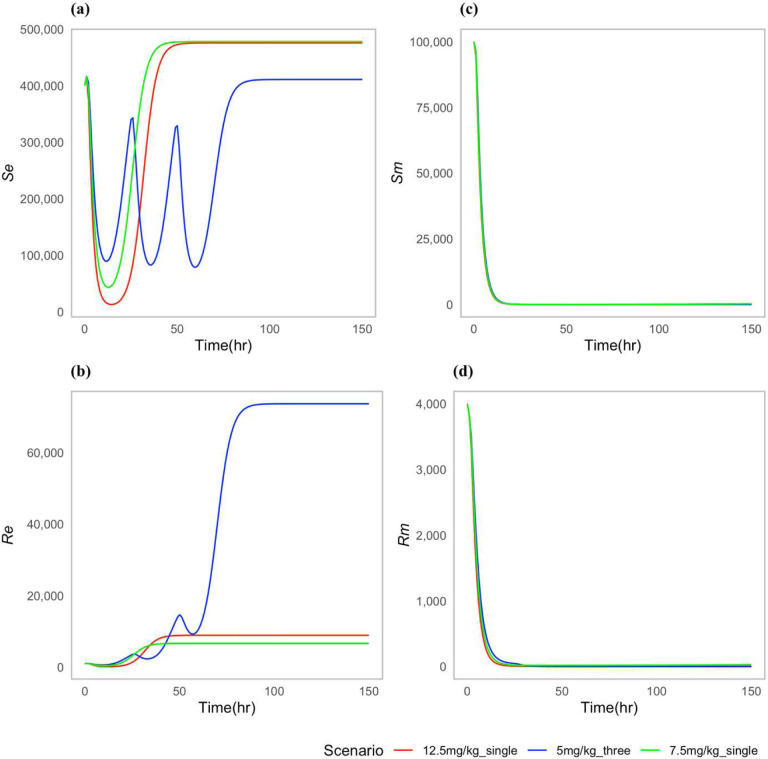
Dynamics of bacterial (*E. coli*) and (*P. multocida*) population under approved dosing scenarios where infection is cleared: (**a**) Susceptible *E. coli* (*S*_*e*_), (**b**) Resistant *E. coli* (*R*_*e*_), (**c**) Susceptible *P. multocida* (*S*_*m*_), (**d**) Resistant *P. multocida* (*R*_*m*_). In this case parameters P= 0.10 and *ϕ*=0.22 used.

**Figure 3:**
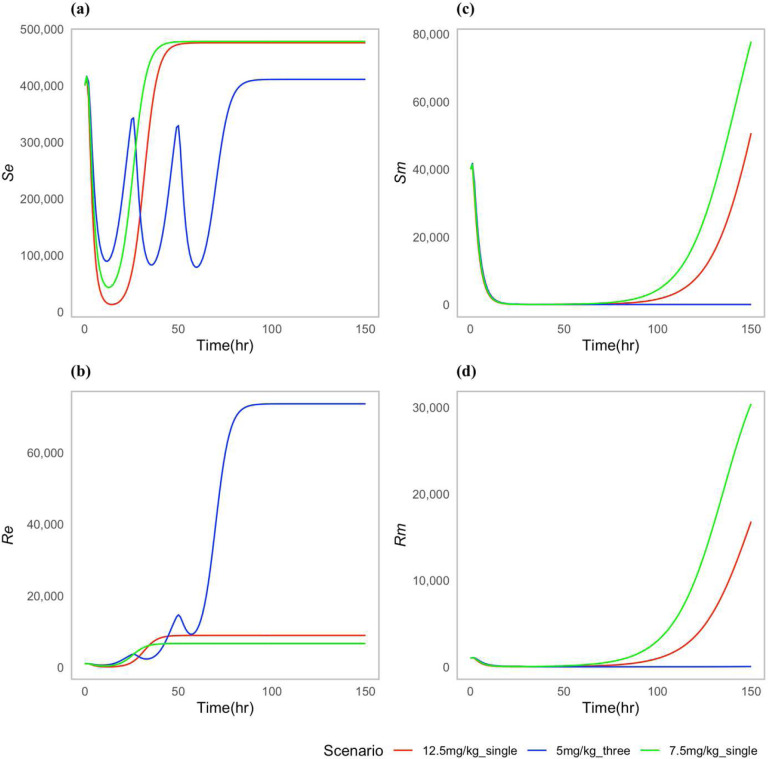
Dynamics of bacterial (*E. coli*) and (*P. multocida*) population under approved dosing scenarios: (**a**) Susceptible *E. coli* (*S*_*e*_), (**b**) Resistant *E. coli* (*R*_*e*_), (**c**) Susceptible *P. multocida* (*S*_*m*_), (**d**) Resistant *P. multocida* (*R*_*m*_).

We choose certain parameters to reflect two types of case scenarios; one in which the approved doses clear the *P. multocida* infection (Fig. 2), and one in which the infection was not cleared for all the approved doses (Fig. 3). For both types of scenarios, the multiple-dose regimen (5 mg/kg, three times) was predicted to clear *P. multocida* infection. While at the assumed resistance level for *P. multocida*, the two single dose regimens failed to clear the infection, and *P. multocida* numbers rebounded after treatment ended. On the contrary, the multiple-dose regimen had a greater effect on the commensal *E. coli* bacteria resistance levels in the gut when compared to the two single dose scenarios (Fig. 3b), indicating the differential antibiotic effect on the different body sites and bacteria populations.

While the two single dose scenarios showed a reduced level of commensal *E. coli* bacteria resistance in the gut (Fig. 3b), they fell short of effectively curing the infection (Fig. 3c,d). Moreover, these two single doses of enrofloxacin administration seemed to correlate with an increase in the prevalence of resistance *P. multocida* bacteria in the lungs (Fig. 3d).

**Figure 4:**
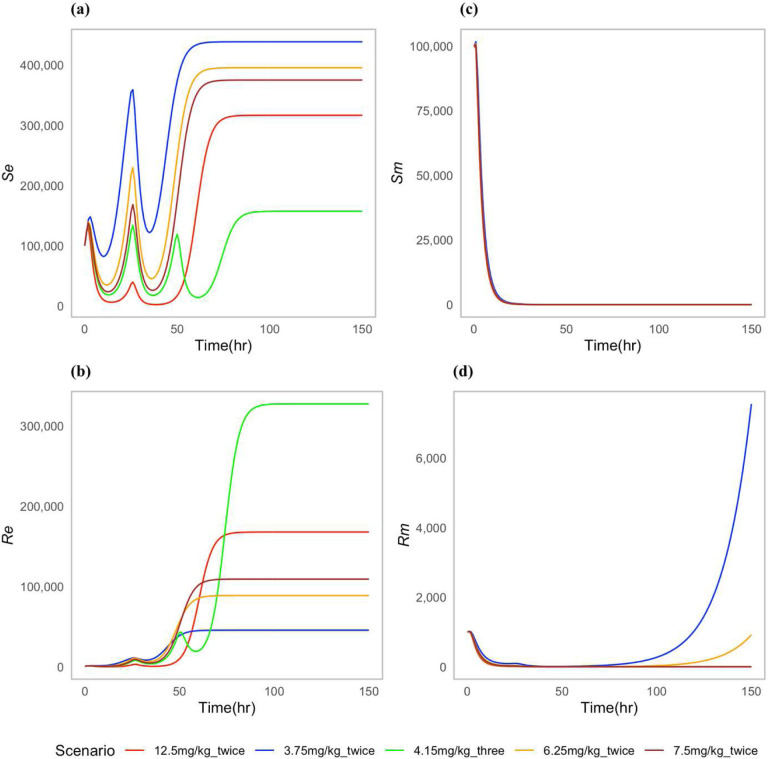
Dynamics of bacterial (*E. coli*) and (*P. multocida*) population under non-approved proposed dosing scenarios: (**a**) Susceptible *E. coli* (*S*_*e*_), (**b**) Resistant *E. coli* (*R*_*e*_), (**c**) Susceptible *P. multocida* (*S*_*m*_), (**d**) Resistant *P. multocida* (*R*_*m*_).

Our study explored some proposed non-approved dosing scenarios by modifying the quantity and frequency of drug administration to the cattle. The goal was to investigate whether there were dose regimens that had similar effects in both the pathogen as well as the off-site bacteria. Trends like the ones in the approved regimes scenarios were observed. The greater the dose and frequency, the larger the resistant *E. coli* population after the treatment (Fig. 4.) Among the tested dose regimens, 3.75 mg/kg administered twice had the greater increase of drug-resistant *P. multocida* bacteria (Fig. 4d). In contrast, a reduced number of resistant commensal *E. coli* bacteria was noted in the gut (Fig. 4b).

When comparing the cost associated to different doses, the 7.5 mg/kg given twice with a 24-hour interval was the most cost effective (Table 3). This treatment cleared the infection within 48 hr and had a moderate effect on the resistant *E. coli* population in the gut (Fig. 4b,d).

### 3.2 Uncertainty and sensitivity analysis result

**Figure 5:**
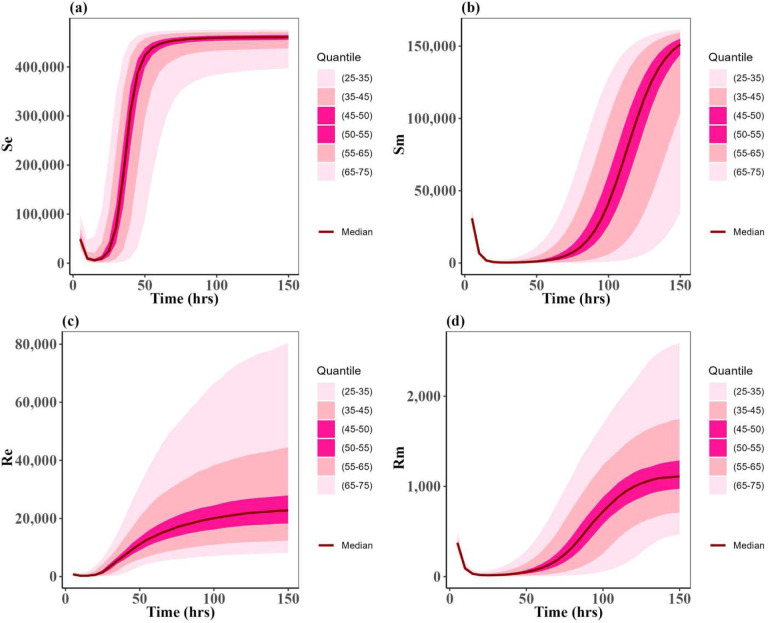
Uncertainty of the states: (**a**) susceptible of *E. coli* (*S*_*e*_), (**b**) susceptible of *P. multocida* (*S*_*m*_), (**c**) resistant of *E. coli* (*R*_*e*_), and (**d**) resistant of *P. multocida* (*R*_*m*_). These distributions for the ODE solutions of model (1) when the parameter set is varied according to the uniform distributions given in Table 2. In this case, the treatment is 12.5 mg/kg single dose. The dark red curves represent the average solution curves (median) of the states obtained from the uncertainty distribution. Other light pink, pink, and dark pink ribbons represent the solutions in 25–75 quantiles of the uncertainty distribution of states.

**Figure 6:**
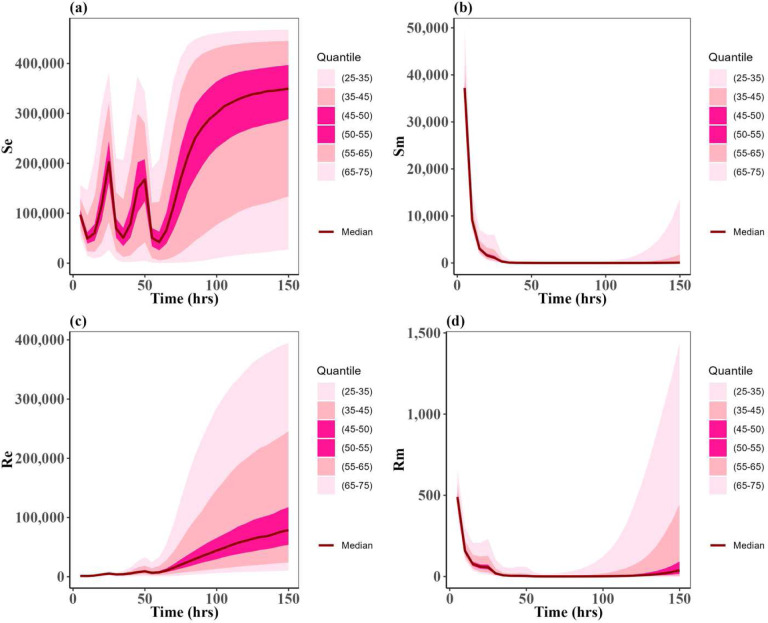
Uncertainty of the states: (**a**) susceptible of *E. coli* (*S*_*e*_), (**b**) susceptible of *P. multocida* (*S*_*m*_), (**c**) resistant of *E. coli* (*R*_*e*_), and (**d**) resistant of *P. multocida* (*R*_*m*_). These distributions are by ODE solution of model (1) for 150 hours having the uniformly distributed parameters input given in the Table 2. The drug dose is 5 mg/kg three times in this case. The dark red curves represent the average solution curves (median) of the states obtained from the uncertainty distribution. Other light pink, pink, and dark pink ribbons represent the solutions in 25–75 quantiles of the uncertainty distribution of states.

Following the algorithm mentioned in the methods section, we simulated the model (1) outcomes for the three approved scenarios for 2000 simulations across the uniform distribution for the PK and PD parameters on Table 2. Figs. 5, and 6 show the uncertainty of each susceptible and resistant bacteria of *E. coli* and *P. multocida* correspondingly in the case of single dose scenario 12 mg/kg and multiple doses scenario 5 mg/kg. As the uncertainty of single dose scenario 7.5 mg/kg shows the identical distribution with single dose scenario 12 mg/kg, we presented the 7.5 mg/kg plot in a supplementary Fig. 1. In this result, we observed the uncertainty of resistant and susceptible *P. multocida* bacteria with the multiple doses scenario 5 mg/kg is low distributed; otherwise, the uncertainty of both bacteria in all other scenarios is highly distributed. As well as the distributed outcomes of the model remain within the range of maximum capacity value of the bacteria populations, denoted as *N*_*emax*_ and *N*_*mmax*_ respectively. These were uniformly distributed parameters as outlined in Table 2. Most importantly, we noted that the median value for resistant and susceptible bacteria of *E. coli* and *P. multocida* matches the dynamic of approved cases presented in Fig. 3. This implies that the lower and upper bounds of the parameters of Table 2 for uniform distribution are one of the optimal sets for the variance-based Sobol sensitivity analysis of the parameters.

**Figure 7:**
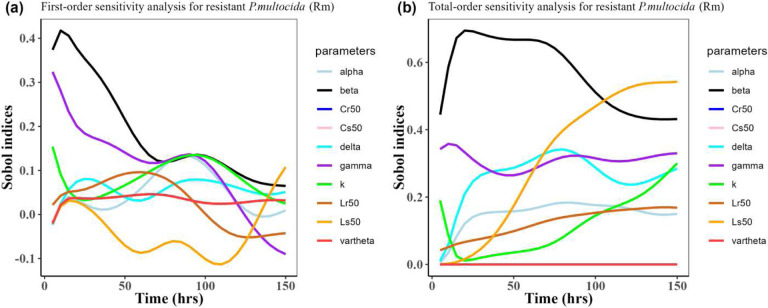
Time-dependent Sobol indices of the set parameters q for the resistance of *P. multocida* (*R*_*m*_) over a time period of 150 hours. In this case, the treatment is 12.5 mg/kg single dose. Some parameters are from the uniform distribution given in Table 2, and others are from Table 1. The Fig. 7**a** shows first-order Sobol indices for *P. multocida* (*R*_*m*_). Likewise, the Fig. 7**b** shows the total-order Sobol indices for *P. multocida* (*R*_*m*_).

**Figure 8:**
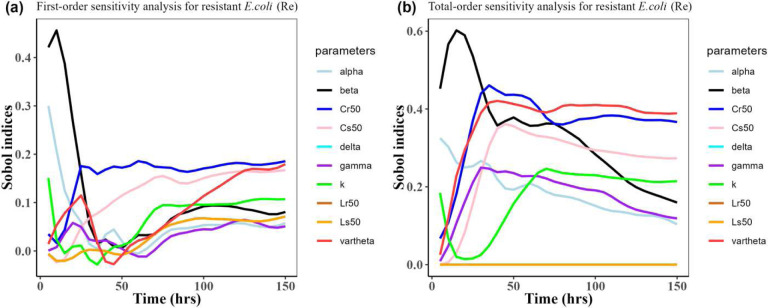
Time-dependent Sobol indices of the set parameters q for the resistance of *E. coli* (*R*_*e*_) over a time period of 150 hours. In this case, the treatment is 12.5 mg/kg single dose. Some parameters are from the uniform distribution given in Table 2, and others are from Table 1. The Fig. 8**a** shows the first-order Sobol indices *E. coli* (*R*_*e*_). Likewise, the Fig. 8**b** shows the total-order Sobol indices for *E. coli* (*R*_*e*_).

**Figure 9:**
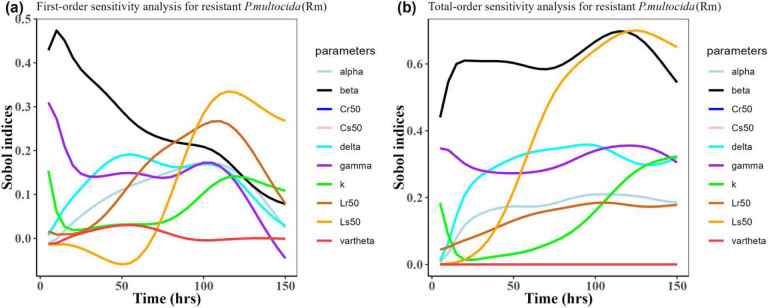
Time-dependent Sobol indices of the set parameters q for the resistance of *P. multocida* (*R*_*m*_) over time period 150 hours. In this case, the drug dose is 7.5 mg/kg single time. Some parameters are from the uniform distribution given in Table 2, and others are from Table 1. The Fig. 9**a** shows the first-order Sobol indices for *P. multocida* (*R*_*m*_). Likewise, the Fig. 9**b** shows the total-order Sobol indices for *P. multocida* (*R*_*m*_).

**Figure 10:**
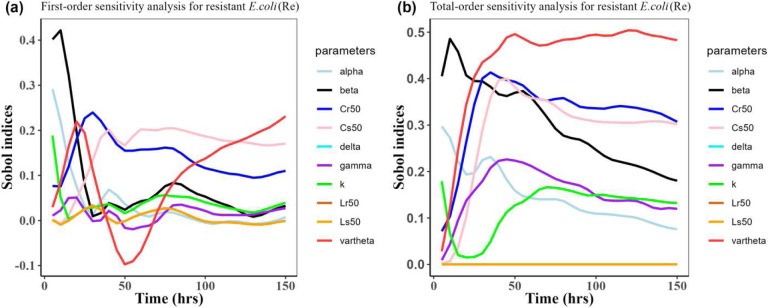
Time-dependent Sobol indices of the set parameters q for the resistance of *E. coli* (*R*_*e*_) over time period 150 hours. In this case, the drug dose is 7.5 mg/kg single time. Some parameters are from the uniform distribution given in Table 2, and others are from Table 1. The Fig. 10**a** is the first-order Sobol indices for *E. coli* (*R*_*e*_). Likewise, the Fig. 10**b** is the total order Sobol indices for *E. coli* (*R*_*e*_).

**Figure 11:**
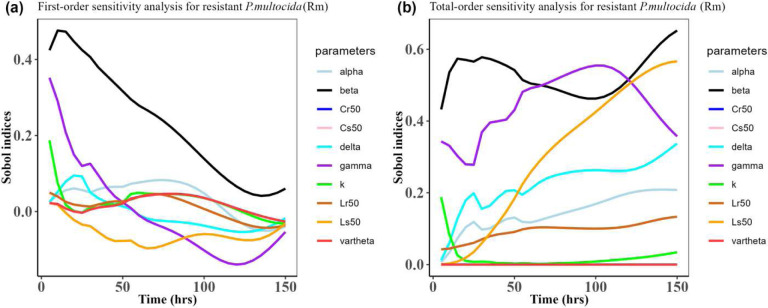
Time-dependent Sobol indices of the set parameters q for the resistance of *P. multocida* (*R*_*m*_) over time period 150 hours. The drug dose is 5 mg/kg three times in this case. Some parameters are from the uniform distribution given in Table 2, and others are from Table 1. The Fig. 11**a** is first-order Sobol indices for the *P. multocida* (*R*_*m*_). Likewise, the Fig. 11**b** is the total-order Sobol indices for *P. multocida* (*R*_*m*_).

**Figure 12:**
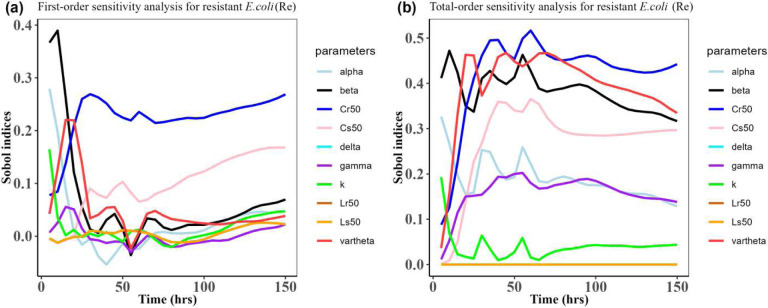
Time-dependent Sobol indices of the set parameters q for the resistance of *E. coli* (*R*_*e*_) over time period 150 hours. The drug dose is 5 mg/kg three times in this case. Some parameters are from the uniform distribution given in Table 2, and others are from Table 1. The Fig. 12**a** is the first-order Sobol indices for *E. coli* (*R*_*e*_). Likewise, the Fig. 12**b** is the total-order Sobol indices for *E. coli* (*R*_*e*_)

Herein, we calculated the sensitivity indices of the PK and PD parameters on Table 2 for the bacterial resistant *R*_*m*_and *R*_*e*_ in the model (1) with all the three approved doses cases scenario (See Figs. 7, 8,9,10,11, and 12). First-order and total-order Sobol indices change with time, making it difficult to decide which parameter the resistant bacteria population is more sensitive to. We then calculated the integrated area of each curve over 150 hours using the Riemann sum method to compare the parameters’ sensitivity levels. The integrated area for all three cases is presented in Tables 4, 5, and 6. The results revealed that the parameter *β*, *υ*, and *C*_*r*50_ are the most sensitive parameters for *R*_*e*_ and *R*_*m*_ in the case of single dose scenario of 12 mg/kg [Table 4]. Similarly, for the single dose scenario 7.5 mg/kg, *β*, *υ*, and *L*_*s*50_ are the most important parameters for the resistant bacteria *R*_*e*_ and *R*_*m*_ [Table 5]. Whereas, in the case of multiple doses scenario 5 mg/kg, we observed that *β*, *γ*, and *C*_*r*50_are most sensitive parameters for *R*_*e*_ and *R*_*m*_ [Table 6].

Overall, the key takeaway from the study of the sensitivity analysis is that *R*_*m*_ and *R*_*e*_ are the most sensitive to the parameters *β*, *υ*, *L*_*s*50_, *C*_*r*50_, and *γ*. Additionally, it is worth noting that the *β* value we estimated matches the one reported by a similar study Erwin et al.^6^.

## 4. Discussion

Antimicrobial use in food animals contributes to the emergence of antimicrobial resistance in commensal gut bacteria and foodborne pathogens that can be transferred to humans through the food chain by direct contact or environmental pathways^34^. The increasing trend of bacterial resistance among food animals has emerged as a growing public health concern^35^. Additionally, resistance in animal pathogens can lead to treatment failures, increased treatment costs, and reduced sale value^36^. Limiting antimicrobial use to the shortest effective duration is crucial for maintaining maximum efficacy while reducing the dissemination of AMR^37^. It is unclear if current dose regimens can both minimize resistance at the site of infection and on off-target bacteria, such as commensal gut bacteria. The interplay between the dosing magnitude, dosing frequency, and bacterial resistance dynamics in both the side of infection and the gut is unknown. Mathematical models can help to elucidate the underlying relationships between the extent of antimicrobial usage and the dynamics of bacterial populations in the gut microbiota^7,38,39^. In this paper, we combined a mathematical model of drug and bacteria dynamics with parameter inference using Pk-Pd modeling tools. This approach enabled us to characterize the uncertainty and its effects on model outcomes.

Our model captures the changes in the bacterial dynamics caused by the competitive release of resistant strains and further selection mediated by enrofloxacin treatment. The dose at which these events have a greater effect in the commensal *E. coli* and *P. multocida* varies. For example, among the approved dosing scenarios, 5 mg/kg as three doses increased the resistance levels in commensal gut enteric *E. coli* bacteria the most. However, the same dose was effective in clearing the *P. multocida* infection and minimizing its resistance. These findings align with prior literature, which has noted that antimicrobial treatment rapidly increases the pool of resistance genes within the gut^40^. Conversely, our investigation revealed that the approved two single dose scenarios to treat BRD infection increased prevalence of resistant pathogenic *P. multocida* bacteria in the lungs in our simulations. This increased prevalence is the result of higher bacterial fitness^41^.

The sensitivity analysis indicated that the model outcomes were particularly sensitive to parameters drug conversion factors into plasma (*β*), elimination of the drug from the colon (*υ*), fifty percent sensitive bacteria (*P. multocida*) killing effect (*L*_*s*50_), fifty percent of bacteria (*E. coli*) above ECOFF killing effect (*C*_*r*50_), and net drug transfer rate in the lung (*γ*).

Our cost analysis considers the most immediate costs associated with treatment failure (i.e, additional treatment, and productivity losses). Our model findings indicate that the two approved single doses treatment scenarios increased overall treatment costs, primarily due to the need to add a treatment round when the infection persists. On the other hand, multiple doses cleared the infection, but they were associated with a cost of a higher number of resistant commensal *E. coli*. The proposed non-approved 7.5 mg/kg administered twice in a 24-hour interval had the lowest cost and overall resistant bacteria. This treatment scenario cleared the infection within a 48-hour time frame and offered the advantage of a lower overall treatment cost. The use of fluoroquinolones in an unapproved manner is illegal in food animals in the United States, so while this regimen may be the most advantageous in this model, we are not advocating for the illegal, extra-label use of enrofloxacin.

Our study was limited by using data to fit the model from experiments conducted on healthy steers. Further research is needed to fully understand how the drug works in animals with respiratory diseases. Additionally, since our data came from healthy animals, we needed more experimental data related to BRD. To address this, we relied on information from existing literature to supplement our model.

## 5. Conclusion

Our findings showed that dose regimens that minimize infection are different from those that minimize resistance in commensal *E. coli* bacteria. Our model’s results underscore the significance of optimizing the currently approved treatment dosing scenarios for treating BRD and using effective antimicrobial treatments in the shortest duration to prevent the spread of AMR.

## Figures and Tables

**Table 1:** Parameter values from literature, calibration, and estimation. Here, ^a^indicates that parameters value used to clear the infection scenario.

Parameters	Description	Values	Units	Source
*k*	Drug absorption rate into plasma	0.44	*hr* ^−1^	Erwin et al.,2020^6^
*β*	Drug conversion factor into plasma	0.0016	*μgmg*^−1^·*kgmL*^−1^	estimated
*α*	Net drug transfer rate in colon	0.16	*hr* ^−1^	estimated
*υ*	Elimination of drug from colon	0.53	*hr* ^−1^	estimated
*σ*	*E. coli* growth rate	0.28	*hr* ^−1^	estimated
*N*_*emax*_	Maximum bacteria (*E. coli*) concentration	484577	*CFU*_*s*_ *g* ^−1^	estimated
*C*_*s*50_	Fifty percent of sensitive bacteria (*E. coli*) killing effect	0.25	*μgmL* ^−1^	Liu et al.,2021^27^
*C*_*r*50_	Fifty percent of bacteria (*E. coli*) above ECOFF bacteria killing effect	0.471	*μgmL* ^−1^	calibrated
*d*	Death rate of *E. coli*	1.01	*hr* ^−1^	calibrated
*c*	Fitness cost of *E. coli*	0.05		calibrated
*γ*	Net drug transfer rate in lung	0.16	*hr* ^−1^	estimated
*λ*	*P. multocida* growth rate	0.25	*hr* ^−1^	calibrated
*N*_*mmax*_	Maximum bacteria (*P. multocida*) concentration	512861	*CFU*_*s*_ *g* ^−1^	calibrated
*L*_*s*50_	Fifty percent of sensitive bacteria (*P. multocida*) killing effect	0.016	*μgmL* ^−1^	Bello et al.,2019^19^
*L*_*r*50_	Fifty percent of bacteria (*P. multocida*) above ECOFF bacteria killing effect	0.125	*μgmL* ^−1^	Bello et al.,2019^19^
*δ*	Elimination of drug from lung	0.24	*hr* ^−1^	estimated
*η*	Death rate of *P. multocida*	0.40	*hr* ^−1^	calibrated
*p*	Fitness cost of *P. multocida*	0.05, 0.10^a^		calibrated
*ϕ*	Immunological death rate	0.14, 0.22^a^	*hr* ^−1^	calibrated

**Table 2:** The lower and upper bound of the set of parameters q, which are applied to perform the Sobol sensitivity analysis in the ‘Sensobal package’ of RStudio. We iterated the simulation 2000 times and used a uniform distribution of each parameter of set q.

Parameters (q)	Lower bound	Upper bound
*k*	0.04	0.9
*β*	0.0001	0.005
*α*	0.01	0.5
*γ*	0.01	0.5
*υ*	0.05	0.9
*δ*	0.07	0.7
*C*_*s*50_	0.2	0.45
*C*_*r*50_	0.3	0.9
*L*_*s*50_	0.006	0.06
*L*_*r*50_	0.05	0.13

**Table 3:** Cost and infection outcomes associated with different treatment scenarios. Here, ^a^indicates that infection was not clear, and bacteria were still present.

Dosing scenario	Time required to cure infection (hrs.)	Accumulative resistant *E. coli* (%)	Treatment cost ($)	Additional treatment if uncured ($)	Total cost ($)
12.5 mg/kg-once	N/A^a^	1.84	43	43	86
6.25mg/kg- twice	52	18.35	48	0	48
5 mg/kg-three	53	15.01	46	0	46
7.5 mg/kg-twice	48	22	41	0	41
12.5 mg/kg-twice	45	34.65	64	0	64
4.15 mg/kg-three	47	67.56	69	0	69
7.5 mg/kg-once	N/A^a^	1.37	37	37	74
3.75 mg/kg-twice	N/A^a^	9.45	42	42	84

**Table 4:** Area obtained by integrating the Sobol indices curves over 150 hours when drug dose is 12.5 mg/kg single time.

Integrated area under the indices curve				
Parameters	First-order		Total-order	
	*R*_*m*_	*R*_*e*_	*R*_*m*_	*R*_*e*_
K	11.81	9.67	15.96	24.96
*β*	26.65	14.87	83.06	49.17
*α*	7.49	7.78	22.15	26.89
*γ*	18.24	4.96	44.72	26.17
*υ*	4.96	12.92	1.49 × 10^−10^	54.34
*δ*	8.36	5.57	38.23	1.59 × 10^−9^
*C*_*s*50_	4.96	17.66	2.08 × 10^−10^	38.67
*C*_*r*50_	4.96	23.22	3.87 × 10^−11^	53.06
*L*_*s*50_	8.78	5.57	46.12	8.16 × 10^−9^
*L*_*r*50_	8.31	5.57	18.02	4.79 × 10^−10^

**Table 5:** Area obtained by integrating the Sobol indices curve over time 150 hours when drug dose is 7.5 mg/kg single time.

Integrated area under the indices curve				
Parameters	First-order		Total-order	
	*R*_*m*_	*R*_*e*_	*R*_*m*_	*R*_*e*_
K	10.40	5.23	18.82	17.42
*β*	35.92	10.88	89.66	44.03
*α*	15.89	4.89	24.97	21.30
*γ*	20.16	2.82	45.38	22.89
*υ*	1.56	17.98	2.51 × 10^−10^	64.93
*δ*	19.46	1.61	42.93	6.29 × 10^−10^
*C*_*s*50_	1.56	23.18	3.2 × 10^−10^	42.14
*C*_*r*50_	1.56	20.34	8.65 × 10^−11^	48.07
*L*_*s*50_	22.05	1.61	60.80	6.78 × 10^−10^
*L*_*r*50_	21.32	1.61	20.91	2.27 × 10^−10^

**Table 6:** Area obtained by integrating the Sobol indices curve over time 150 hours when drug dose is 5 mg/kg three times.

Integrated area under the indices curve				
Parameters	First-order		Total-order	
	*R*_*m*_	*R*_*e*_	*R*_*m*_	*R*_*e*_
K	4.51	2.67	2.38	5.41
*β*	33.46	8.84	76.77	55.49
*α*	7.92	5.30	20.99	27.26
*γ*	14.45	2.13	65.31	23.31
*υ*	3.37	7.53	1.10 × 10^−10^	57.81
*δ*	5.66	1.54	32.86	5.42 × 10^−10^
*C*_*s*50_	3.37	14.57	1.08 × 10^−10^	39.78
*C*_*r*50_	3.37	32.66	7.18 × 10^−11^	61.61
*L*_*s*50_	9.05	1.54	44.52	4.36 × 10^−10^
*L*_*r*50_	4.12	1.54	13.72	4.76 × 10^−10^

## Data Availability

The datasets generated and/or analyzed during the current study are available from the corresponding author on request.
